# An adjusted Asia-Pacific colorectal screening score system to predict advanced colorectal neoplasia in asymptomatic Chinese patients

**DOI:** 10.1186/s12876-023-02860-x

**Published:** 2023-06-29

**Authors:** Chenchen Zhang, Liting Zhang, Weihao Zhang, Bingxin Guan, Shuai Li

**Affiliations:** 1 Department of Gastroenterology, the Second Hospital, Cheeloo College of Medicine, Shandong University, Beiyuan Street & 247, Jinan, 0531 Shandong China; 2 Department of Gastrointestinal Endoscopy Center, the Second Hospital, Cheeloo College of Medicine, Shandong University, Jinan, China; 3 Department of Pathology, the Second Hospital, Cheeloo College of Medicine, Shandong University, Jinan, China

**Keywords:** Advanced colorectal neoplasia, Colorectal cancer screening, Risk stratification, Scoring system

## Abstract

**Purpose:**

The Asia-Pacific Colorectal Screening (APCS) score and its derivatives have been used to predict advanced colorectal neoplasia (ACN). However, it remains unknown whether they apply to the current Chinese population in general clinical practice. Therefore, we aimed to update the APCS score system by applying data from two independent asymptomatic populations to predict the risk of ACN in China.

**Methods:**

We developed an adjusted APCS (A-APCS) score by using the data of asymptomatic Chinese patients undergoing colonoscopies from January 2014 to December 2018. Furthermore, we validated this system in another cohort of 812 patients who underwent screening colonoscopy between January and December 2021. The discriminative calibration ability of the A-APCS and APCS scores was comparatively evaluated.

**Results:**

Univariate and multivariate logistic regression were applied to assess the risk factors for ACN, and an adjusted scoring system of 0 to 6.5 points was schemed according to the results. Utilizing the developed score, 20.2%, 41.2%, and 38.6% of patients in the validation cohort were classified as average, moderate, and high risk, respectively. The corresponding ACN incidence rates were 1.2%, 6.0%, and 11.1%, respectively. In addition, the A-APCS score (c-statistics: 0.68 for the derivation and 0.80 for the validation cohort) showed better discriminative power than using predictors of APCS alone.

**Conclusions:**

The A-APCS score may be simple and useful in clinical applications for predicting ACN risk in China.

## Introduction

Colorectal cancer (CRC) accounts for approximately 10.0% of all confirmed cancers and cancer-related mortality worldwide [1]. The 5 year cumulative survival rate of CRC patients by tumor staging was listed as follows: stage 0, 94.0%; stage I, 91.6%; stage II, 84.8%; stage IIIa, 77.7%; stage IIIb, 60.0%; and stage IV, 18.8%, which implies that patients could obtain survival advantage if the tumors were treated by en bloc resection with confirming negative margins in the earliest stage [2]. Colonoscopy is currently the preferred method for CRC diagnosis. With the help of mucosal staining and magnifying endoscopy, precancerous lesions can be detected by the naked eye. Once diagnosed, endoscopic polypectomy can effectively remove malignant polyps [3]. Therefore, screening for CRC in healthy asymptomatic individuals is essential, and several countries have formulated population-based screening approaches. The incidence of CRC strongly increases with age; it is low before 50 and shows a rapid upward trend after 50. However, the incidence increased rapidly from the age of 40 in East Asia. As a result, the starting age for CRC screening is over 40 years in China and Japan [2], whereas in European and American countries, it is often over 45 years [4, 5].

Considering that the limited capacity of colonoscopy hinders the implementation of CRC screening in many countries, it is worthwhile to establish a risk-stratification system to make screening more cost-effective. Combined risk factors and patient characteristics for stratification are considered valuable, and multiple CRC screening risk-stratification systems have already been formulated on request [6–14]. Among these, the Asia-Pacific Colorectal Screening (APCS) score has been validated for predicting advanced colorectal neoplasia (ACN). The APCS score is based on four factors: age, sex, family history of CRC, and smoking [8]. The modified APCS score system incorporates body mass index (BMI) as a new predictor of APCS score [15]. A recent Japanese study regulated the modified APCS score from a 6-point to an 8-point system for the Japanese population, which improved the discriminative ability of the score [16]. However, the new scoring model used to assess the risk-stratification of outpatients is somewhat complex, as it requires knowledge of exactly how much and for how long a patient has smoked. In addition, the APCS score is based on data across nine ethnic populations; hence, a deeper regionalization study is required in different countries, considering the heterogeneity of population characteristics. Third, all previously reported scores used data from patients 10 or 20 years ago; therefore, the discriminative abilities for current population risk-stratification are unknown. Therefore, further adjustment and verification are essential for the clinical application of the APCS score in CRC screening. Our objective was to establish and examine a new scoring system based on the APCS for predicting ACN risk in China by applying data from two independent asymptomatic populations.

## Methods

### Study population

We conducted a retrospective survey at the Second Hospital, Cheeloo College of Medicine, Shandong University (Shandong, China) between January 2014 and December 2018, and the patients were included in the derivation cohort for the development of the scoring model. Another independent group of patients, who attended to the hospital between January and December 2021, was prospectively enrolled for the model validation (validation cohort). Patients older than 40 years who underwent screening colonoscopy with no symptoms of the lower digestive tract were included. Exclusion criteria included a personal history of inflammatory bowel disease, hereditary polyposis syndromes (familial adenomatous polyposis and hereditary nonpolyposis CRC), inadequate colon preparation, and lack of detail of medical history or BMI.

This study followed the ethical standards formulated in the Helsinki Declaration and was approved by the ethics committee of the Second Hospital, Cheeloo College of Medicine, Shandong University (ethical approval number: KYLL-2022LW048). Furthermore, all personally identifiable information had already been de-identified to maintain patient privacy; therefore, the research program was exempt from the participants’ informed consent requirement.

### Study definitions and data collection

Patient information, including BMI, demographics, family history of CRC in first-degree relatives (FDR), colonoscopy findings, and pathology results, were gathered from electronic medical records. Current or past smokers were defined as those who smoked seven or more cigarettes per week and current or past drinkers as those who consumed alcohol ≥ 2 times/week. Oral administration of aspirin or metformin twice or more weekly over 12 months was defined as regular intake of aspirin/metformin [17].

**Fig. 1:**
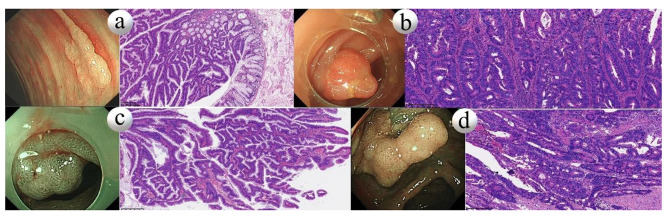
Representative endoscopic picture (left) and pathological picture of advanced colorectal neoplasia (right). (a) 1.0 × 0.6 cm tubular adenoma (Left, narrow band imaging; Right, × 40 magnification). (b) 0.6 × 0.5 cm tubular adenoma with HGD (Left, white light image; Right, × 100 magnification). (c) 0.5 × 0.5 cm tubulovillous adenoma (Left, narrow band imaging; Right, × 40 magnification). (d) 2.5 × 1.5 cm tubular adenocarcinoma (Left, narrow band imaging; Right, × 100 magnification)

Adenomas were classified into three classes according to size: diminutive (1–5 mm in diameter), small (6–9 mm), and large (≥ 10 mm) [18]. Advanced neoplasia was defined as an advanced adenoma (an adenoma with a villous or tubulovillous component, high-grade dysplasia [HGD], or size ≥ 10 mm) or invasive carcinoma (shown in Fig. 1). In addition, sessile serrated lesions (SSLs) with diameters ≥ 10 mm or HGDs were classified as advanced neoplasia and diminutive or small SSLs were classified non-neoplastic.

Colonoscopies were performed using standard colonoscopes (CF Q260AI, CF H260AI, PCF-Q260AZI, CF-HQ290ZI, or PCF-H290ZI; OLYMPUS, Tokyo, Japan) by three experienced endoscopists. Pathological diagnoses were interpreted and confirmed by expert pathologists. Cecal intubation involved the colonoscope tip passing the ileocecal valve to the appendicular stoma [19]. The rating scale for bowel preparation was assessed using the Boston bowel preparation scale, and “adequate” was defined as a score of ≥ 2 for each segment. The adenoma detection rate was defined as the percentage of patients who had ≥ 1 conventional adenoma detected on the first-time primary colonoscopy.

### Calculation and validation of the risk score

Univariate and multivariate logistic regression analyses were performed to determine the association between ACN and each risk factor in the derivation cohort. Risk factors included age, sex, smoking, alcohol consumption, aspirin/metformin intake, BMI, and history of FDR with CRC. Based on the multivariate analysis results, each independent risk factor was assigned a weight, applying the corresponding odds ratio (OR) halved, and then rounded off after the first decimal point in units of 0.5 [8]. The total score for each patient was the sum of the scores of each risk factor, and patients were then classified into three separate groups (average risk [AR], moderate risk [MR], and high risk [HR]) according to the distribution of ACN. After the adjusted APCS (A-APCS) score was formulated, its discriminatory ability was tested in the derivation and validation cohorts.

### Statistical analysis

Descriptive statistics were presented as numbers and percentages, and used to tabulate the characteristics of the screening population and clinicopathological features of advanced neoplasia in patients enrolled in the study, which were compared using chi-square tests. The associations between ACN and personal data of screened patients were determined by univariate analyses using the chi-square test or Fisher’s exact test. The BMI threshold was based on the result of the receiver operating characteristic (ROC) test. The figure maximizing the sum of the sensitivity and specificity for ACN was set as the cut-off value. Significant variables (P < 0.2) in the univariate analysis were included in the multiple analyses to set the independent risk factors. Odds ratios (OR) and 95% confidence intervals (CI) for each factor was calculated for the following weight setting. The goodness-of-fit index was examined using the Hosmer–Lemeshow goodness-of-fit test and a p > 0.05 implied a good fitness of forecast risk against the actual risk. As mentioned above, the A-APCS score for predicting ACN in the Chinese population was formulated based on the identified risk factors. Next, the ACN distribution according to subgroups in each cohort was calculated. The ROC curve and c-statistics were used to detect the potency of the A-APCS score in predicting ACN. The discriminative power of the APCS score was also detected using c-statistics, and the DeLong test was used to compare the value with that of the A-APCS score. Cohen’s kappa Statistic was employed to measure the level of agreement between the two scoring systems. Statistical analyses were performed using the SPSS (version 25.0; IBM Corp, Armonk, NY) and MedClac version 20.0 (MedCalc Software Ltd, Ostend, Belgium), and statistical significance was set at 0.05.

## Results

### Baseline features of screening patients included in the study

**Fig. 2:**
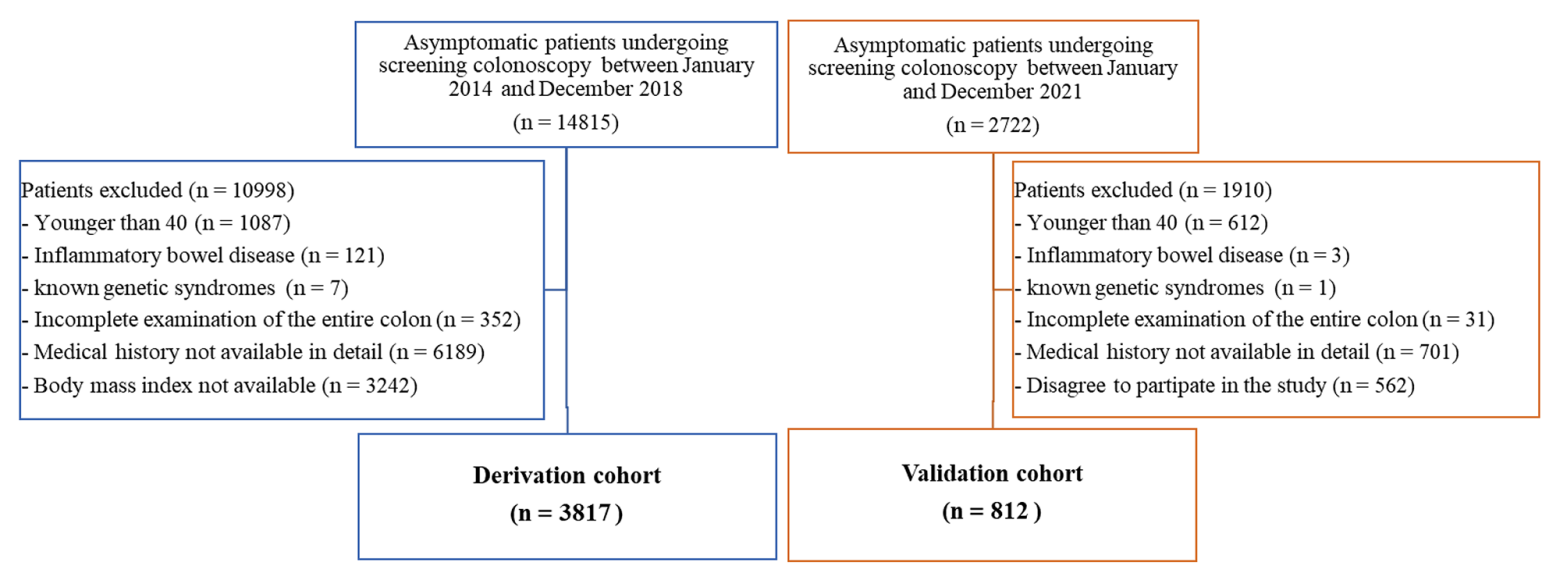
Flow chart of study participants

A total of 14,815 patients underwent screening colonoscopy between January 2014 and December 2018, of which 3817 were recruited in the derivation cohort. Further, 812 out of 2722 patients, who attended to the hospital between January and December 2021, were included in the validation cohort according to the inclusion and exclusion criteria (shown in Fig. 2). The detailed features of all the patients are listed in Table 1. The characteristics of the Derivation and Validation cohorts were similar according to age, sex, aspirin or metformin intake, history of FDR with CRC, and proportion of patients with colorectal neoplasia (all p > 0.05). However, in the derivation cohort, the rates of smoking, drinking, and BMI ≥ 23.5 kg/m^2^ were higher than that in the validation cohort (all P *<* 0.05). Regarding quality indicators, a high cecal intubation rate and adequate bowel preparation in both cohorts guaranteed the quality of colorectal cancer screening. The adenoma detection rates in the derivation and validation cohorts were 22.6% and 16.1%, respectively (P = 0.000). The clinicopathological features of ACN detected across the study are shown in Table 2 and were similar in the two cohorts stratified by pathology and location (all p > 0.05).

**Table 1:** Features of screening patients included in the study

	Derivation cohort (N = 3817)	Validation cohort (N = 812)	P
Age (years)			0.103
≤ 49, n (%)	753 (19.7)	159 (19.6)	
50‒69, n (%)	2740 (71.8)	602 (74.1)	
≥ 70, n (%)	324 (8.5)	51 (6.3)	
Sex, male, n (%)	2405 (63.0)	509 (62.7)	0.863
Smoking, n (%)	1887 (49.4)	259 (31.9)	**0.000**
Alcohol consumption, n (%)	1503 (39.4)	234 (28.8)	**0.000**
Intake of aspirin, n (%)	124 (3.2)	37 (4.6)	0.065
Intake of metformin, n (%)	81 (2.1)	19 (2.3)	0.698
BMI (kg/m^2^)			
< 23.5, n (%)	1722 (45.1)	423 (52.1)	**0.000**
≥ 23.5, n (%)	2095 (54.9)	389 (47.9)	
History of FDR with CRC, n (%)			0.820
None	3463 (90.7)	733 (90.3)	
One	331 (8.7)	75 (9.2)	
Two or more	23 (0.6)	4 (0.5)	
Patients with colorectal neoplasia, n (%)			
ACN	267 (7.0)	42 (5.2)	0.059
CRC	37 (1.0)	5 (0.6)	0.335
Quality indicators, n (%)			
Cecal intubation rate	3538 (92.7)	766 (94.3)	0.096
Adequate bowel preparation	3586 (93.9)	766 (94.3)	0.673
Adenoma detection rate	863 (22.6)	131 (16.1)	**0.000**

Significant P values are shown in bold text

Abbreviations: ACN, advanced colorectal neoplasia; BMI, body mass index; CRC, colorectal cancer; FDR, first-degree relative.

**Table 2:** Clinicopathological features of advanced neoplasia detected in the study

	Derivation cohort (N = 294) n (%)	Validation cohort (N = 50) n (%)	P
Pathology			0.918
Colorectal cancer	37 (12.6)	5 (10.0)	
Diminutive and small adenoma			
With high-grade dysplasia	70 (23.8)	10 (20.0)	
With ≥ 25% villous features	16 (5.4)	4 (8.0)	
Large adenoma			
With no advanced histology	53 (18.0)	11 (22.0)	
With high-grade dysplasia	103 (35.0)	18 (36.0)	
With ≥ 25% villous features	15 (5.1)	2 (4.0)	
Location			0.986
Proximal colon	118 (40.1)	20 (40.0)	
Distal colon	176 (59.9)	30 (60.0)	

Note: Adenomas with high-grade dysplasia and ≥ 25% villous features are classified into the high-grade dysplasia group

### Univariate and multivariate predictors of ACN in the derivation cohort

Univariate and multivariate analyses of the associations between the features of the screened patients and ACN are tabulated in Table 3. We adopted a cut-off value of 23.5 kg/m^2^ for BMI based on the results of the ROC analysis. In the univariate analysis, higher age, male sex, current or past smoking, BMI ≥ 23.5 kg/m^2^, and one or more FDRs with CRC were closely correlated with ACN (all P < 0.05). We enrolled these five variables in the multivariate analysis as well as alcohol consumption with P < 0.2. Finally, the independent factors were higher age (50‒69 years: OR, 2.0; 95% CI, 1.2‒3.3; ≥ 70 years: OR, 3.8, 95% CI, 2.3‒6.4), male sex (OR, 2.0; 95% CI, 1.4‒3.0), current or past smoking (OR, 1.4; 95% CI, 1.0‒2.0), BMI ≥ 23.5 kg/m^2^ (OR, 1.7; 95% CI, 1.3‒2.3), and family history of CRC in FDRs ( OR, 1.7; 95% CI, 1.1‒2.4; two or more: OR, 3.8, 95% CI, 1.4‒10.8). The Hosmer‒Lemeshow goodness-of-fit test exhibited P = 0.376 for the derivation cohort, which implied a good match between forecast risk and actual risk.

**Table 3:** Univariate and multivariate analysis of clinical features of screening patients in Derivation cohort related to advanced colorectal neoplasia

Characteristics	Advanced colorectal neoplasia, n (%)		Univariate analysis		Multivariate analysis	
	Present (N = 267)	Absent (N = 3550)	Odds ratio (95% CI)	P	Odds ratio (95% CI)	P
Age (years)				**0.000**		
40‒49	29 (10.9)	724 (20.4)	1		1	
50‒69	202 (75.7)	2538 (71.5)	2.0 (1.3‒3.0)		2.0 (1.2‒3.3)	**0.001**
≥ 70	36 (13.5)	288 (8.1)	3.1 (1.9‒5.2)		3.8 (2.3‒6.4)	**0.000**
Sex				**0.000**		
Women	50 (18.7)	1362 (38.4)	1		1	
Men	217 (81.3)	2188 (61.6)	2.7 (2.0‒3.7)		2.0 (1.4‒3.0)	**0.000**
Smoking				**0.000**		
No	87 (32.6)	1843 (51.9)	1		1	
Current or past	180 (67.4)	1707 (48.1)	2.2 (1.7‒2.9)		1.4 (1.0‒2.0)	**0.028**
Alcohol consumption				0.072		
No	148 (55.4)	2166 (61.0)	1			
Current or past	119 (44.6)	1384 (39.0)	1.3 (1.0‒1.6)			0.346
Intake of aspirin				0.339		
Present	6 (2.2)	118 (3.3)	1			
Absent	261(97.8)	3432(96.7)	1.5 (0.7‒3.4)			
Intake of metformin				0.240		
Present	3 (1.1)	78 (2.2)	1			
Absent	264 (98.9)	3472 (97.8)	2.0 (0.6‒6.3)			
BMI (kg/m^2^)				**0.000**		
< 23.5	82 (30.7)	1640 (46.2)	1		1	
≥ 23.5	185 (69.3)	1910 (53.8)	1.9 (1.5‒2.5)		1.7 (1.3‒2.3)	**0.000**
History of FDR with CRC				**0.000**		
None	227 (85.0)	3236 (91.2)	1		1	
One	35 (13.1)	296 (8.3)	1.7 (1.2‒2.5)		1.7 (1.1‒2.4)	**0.009**
Two or more	5 (1.9)	18 (0.5)	4.0 (1.5‒10.8)		3.8 (1.4‒10.8)	**0.011**

Significant P values are shown in bold text

BMI, body mass index; CI, confidence interval; CRC, colorectal cancer; FDR, first-degree relative

### Development of the adjusted Asia-Pacific Colorectal Screening (A-APCS) score

According to the OR identified in the multivariate analysis, the following risk factors were considered to give points: age 40‒49 (0), 50‒69 (1), ≥ 70 years (2); female sex (0), male sex (1); no smoking (0), current or past smoking (0.5); BMI < 23.5 kg/m^2^ (0), BMI ≥ 23.5 kg/m^2^ (1); FDR with CRC none (0), one (1), two or more (2). The new scoring model varied between 0 and 6.5 (Table 4), in contrast to 0–7 of APCS score (Table 5). When scoring all patients using the A- APCS score and APCS score, Cohen’s Kappa value turns out to be 0.234 and 0.177 for the Derivation and Validation cohorts, respectively.

**Table 4:** Adjusted Asia-Pacific Colorectal Screening score for advanced colorectal neoplasia

Risk factor	Criteria	Points
Age (years)	40‒49	0
	50‒69	1
	≥ 70	2
Sex	Women	0
	Men	1
Smoking	No	0
	Current or past	0.5
BMI (kg/m^2^)	< 23.5	0
	≥ 23.5	1
History of FDR with CRC	None	0
	One	1
	Two or more	2

Abbreviations: BMI, body mass index; CRC, colorectal cancer; FDR, first-degree relative

**Table 5:** Asia-Pacific Colorectal Screening score for advanced colorectal neoplasia

Risk factor	Criteria	Points
Age (years)	40‒49	0
	50‒69	2
	≥ 70	3
Sex	Women	0
	Men	1
Smoking	No	0
	Current or past	1
History of FDR with CRC	None	0
	One or more	2

Abbreviations: CRC, colorectal cancer; FDR, first-degree relative

### Risk stratification of the study population assessed with the A- APCS score

**Fig. 3:**
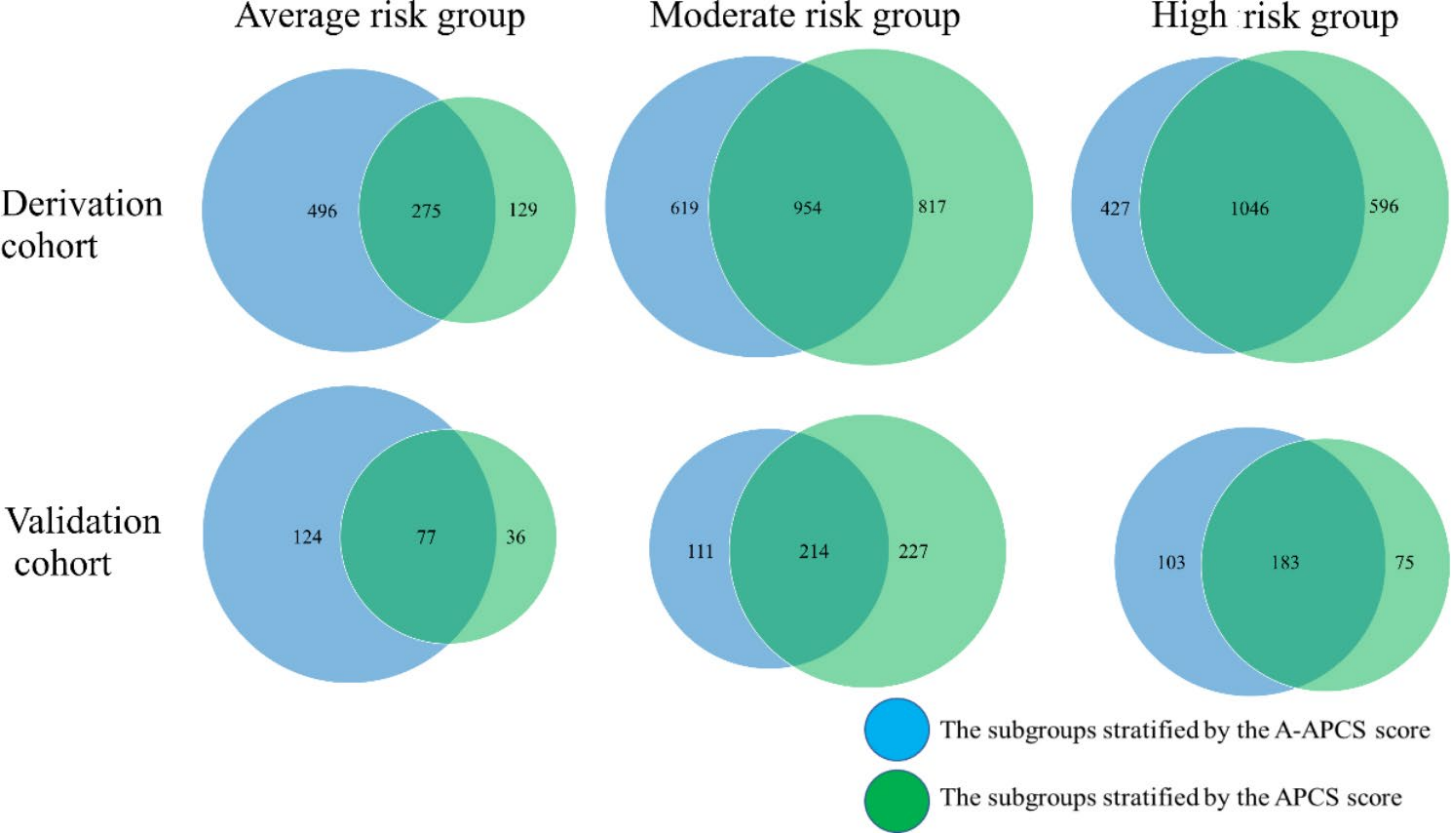
Distribution of the study population in the Derivation (N = 3817) and Validation cohorts (N = 812) based on the risk stratifications by two scoring systems

**Fig. 4:**
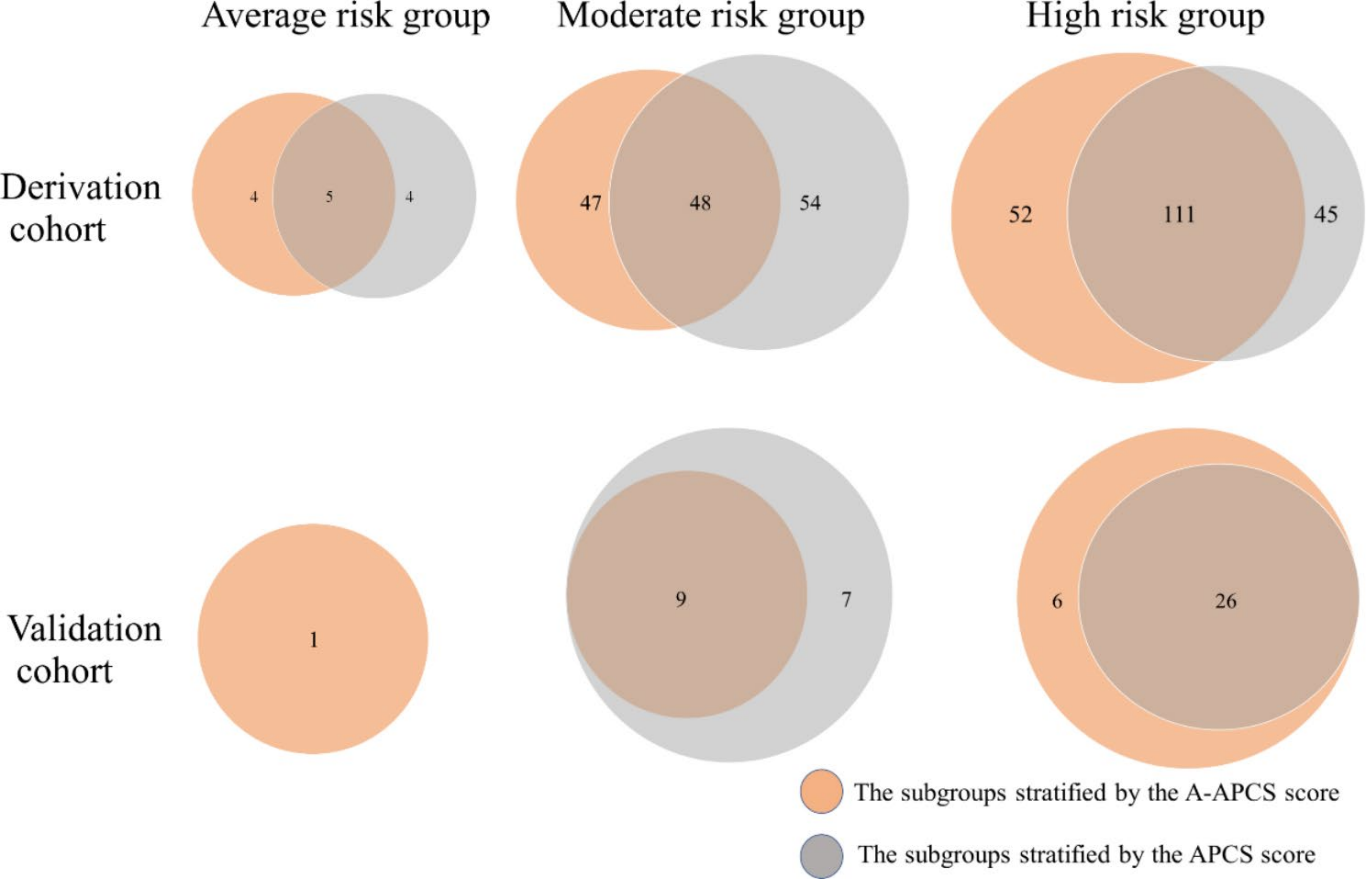
Distribution of patients with advanced colorectal neoplasia in the Derivation (N = 267) and Validation cohorts (N = 42) based on the risk stratifications by two scoring systems

Using the A-APCS score, the study population was divided into three subgroups: AR, score 0 to 1; MR, score 1.5 to 2.5; and HR, score 3‒6.5 (Table 6). In the derivation cohort, 771 (20.2%), 1573 (41.2%), and 1473 (38.6%) patients were in the AR, MR, and HR groups, respectively. The prevalence of ACN in the three categories was 1.2% (95% CI, 0.4‒1.9), 6.0% (95% CI, 4.9‒7.2), and 11.1% (95%CI, 9.5‒12.7), respectively. The incidence of ACN in the validation cohort stratified by the A-APCS score was 1 (0.5%), 9 (2.8%), and 32 (11.2%) in the AR, MR, and HR groups, respectively (Table 6). The distributions of the study population and patients with ACN based on the risk stratifications by two scoring systems were shown in Figs. 3 and 4. When assessing the agreement between two scoring systems for risk stratification using Cohen’s Kappa Statistic, the value for the Derivation cohort and Validation cohort was 0.451 and 0.346, respectively.

**Table 6:** Distribution of advanced colorectal neoplasia based on subgroups in the Derivation (N = 3817) and Validation cohorts (N = 812)

Subgroups	Derivation cohort		Validation cohort	
	The proportion of Patients with ACN (%)	95% CI (%)	The proportion of Patients with ACN (%)	95% CI (%)
Average risk (0‒1)	1.2 (9/771)	0.4‒1.9	0.5 (1/201)	-0.4‒1.5
Moderate risk (1.5‒2.5)	6.0 (95/1573)	4.9‒7.2	2.8 (9/325)	1.0‒4.6
High risk (3‒6.5)	11.1 (163/1473)	9.5‒12.7	11.2 (32/286)	7.5‒14.9
Total	7.0 (267/3817)	6.2‒7.8	5.2 (42/812)	3.6‒6.7

Abbreviations: ACN, advanced colorectal neoplasia; CI, confidence interval

### Validity and reliability of the A- APCS score

**Fig. 5:**
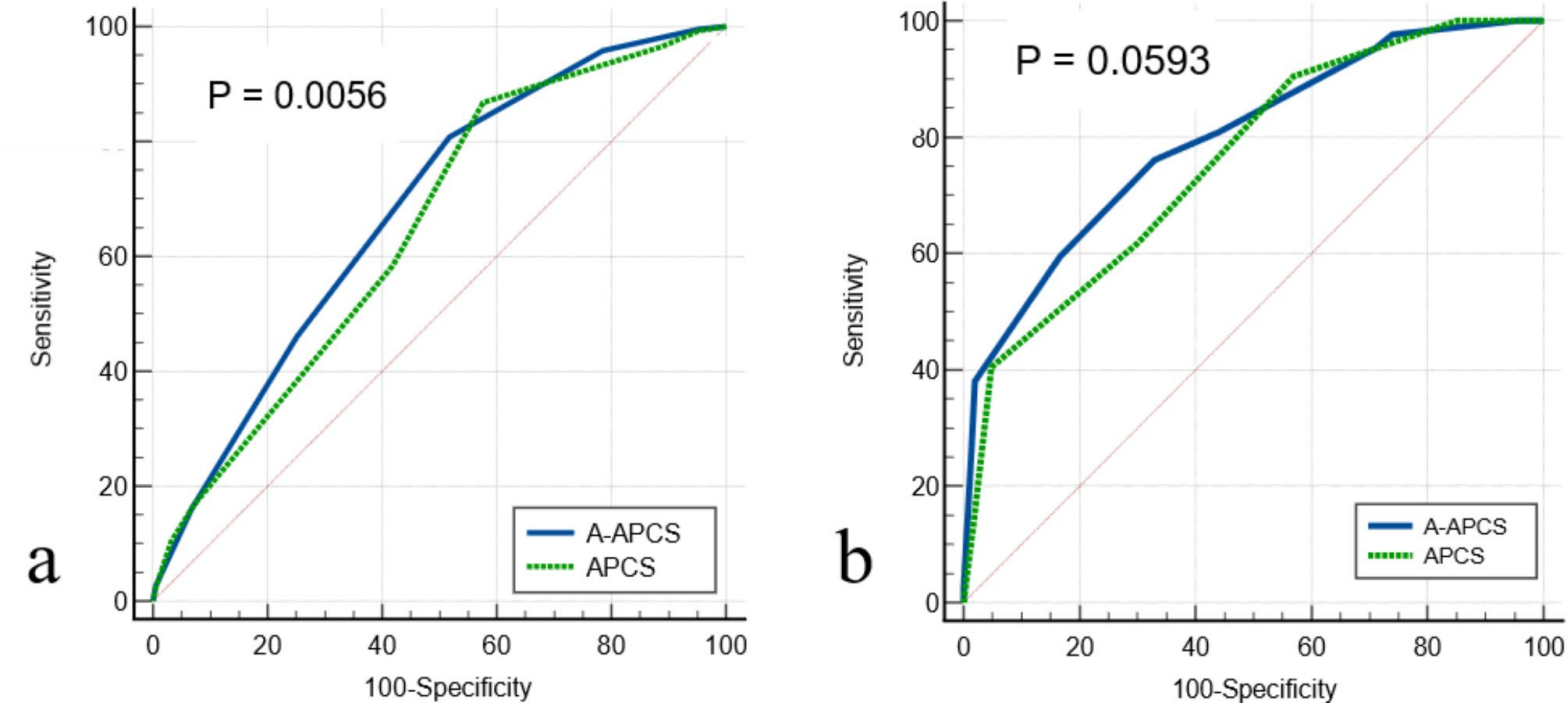
Comparison of c-statistics by the receiver operating characteristic curves to assess the discriminative power of two scoring models for predicting advanced colorectal neoplasia. (a) Derivation cohort. (b) Validation cohort

The c-statistic of the A-APCS score for predicting ACN in the 3817 patients of the derivation cohort was 0.68 (95% CI, 0.66‒0.69), which implied a high discriminating power (shown in Fig. 5). In comparison with the APCS score, the latter (0.65; 95% CI, 0.63‒0.76) was significantly lower (P = 0.0056). Moreover, the c-statistic for risk predictors of A-APCS was 0.80 (95% CI, 0.77‒0.82), which was higher than that for APCS score (0.76; 95% CI, 0.73‒0.79) in the validation cohort; however, the difference was not statistically significant (P = 0.0593).

## Discussion

According to the risk factors for CRC, the Asia-Pacific Working Group on CRC formulated the APCS score to stratify ACN risk in asymptomatic patients in 2011. Several studies modified the scoring system based on the population characteristics in different regions of Asia, and incorporated BMI as an additional variable to improve discriminating power [11, 15, 16, 20, 21]. The APCS score or modified APCS scores have been adopted for CRC screening in parts of the Asia-Pacific region, but it remains unknown whether they apply to the current Chinese population in general clinical practice. In China, the guidelines recommend that CRC screening should start at age 40 for the general population; however, the APCS score was put forward on the version targeting the population aged over 50 years. With social progress, the BMI of Chinese general population is gradually increasing (22.7 kg/m^2^ in 2004 compared to 24.4 kg/m^2^ in 2018) [22, 23], and drinking or smoking habits are changing [24]. In addition, researchers are increasingly finding that regular aspirin or metformin use reduces risk of CRC [3, 25]. Consequently, it would be advisable to formulate an adjusted scoring model for predicting ACN through a dataset of screened patients in recent years, and further, introduce the model for clinical application in China.

The present study clarified the demographic characteristics of the two cohorts at different periods, including various prevalence values of ACN according to clinicopathological features. Further, we built a scoring model for predicting ACN risk in China. Applying a retrospective derivation study (data from 6 to 8 years ago) and a prospective validation study (data from 2 years ago) other than previous prospective studies (data from 10 to 20 years ago) to develop the scoring model ensured the usefulness of risk-stratification for CRC screening in the current Chinese population. Most features in the derivation cohort and the validation set were similar and consistent with other studies [15, 16, 26], showing the model’s reliability. In the current study the prevalence of ACN (7.0%) and CRC (1.0%) in the derivation cohort seems slightly higher than that in many previous studies. This difference could be due to the high-quality endoscopy (magnifying endoscopy with narrow-band imaging, ME-NBI) used in this study by experienced endoscopists in our gastroenterology department. As mentioned in previous studies, ME-NBI can improve the diagnostic accuracy of colorectal neoplasia, resulting in differences in lesion detectability [27, 28]. Moreover, a high cecal intubation rate and adequate bowel preparation guaranteed high detectability, particularly in detecting diminutive and small adenomas. Under these conditions, the quality of the ACN data in this study is credible.

The weightage assigned to smoking was lower than the APCS or modified APCS score (0.5 in present study compared to 1 in the APCS or modified APCS score). In the multivariate logistic regression analysis for ACN in the APCS and modified APCS models, the odds ratios for smoking were 1.8 (P = 0.099) and 1.63 (P = 0.026) respectively, as compared to 1.4 (P = 0.028) in the present study. There are several possible explanations for this discrepancy. First, the heterogeneity among Asian populations could be a possible factor. The APCS study enrolled patients from 11 Asian cities, while the modified APCS study recruited only the population in Hong Kong. In contrast, the present study exclusively included Chinese patients in Shandong. Second, research has argued that smoking increases the risk of CRC in a dose-dependent manner [29, 30]. However, for the past 10 years, smokers have attempted to quit smoking in pursuit of a healthy lifestyle using behavioral therapy, nicotine patches, chewing gum, and medicines [31]. Finally, the scientific and technological revolution, such as the types of tobacco and cigarette production technology processing, have critical effects on the carcinogens that can affect the colorectal mucosa. Based on the ROC analysis, we used a cut-off level of 23.5 kg/m^2^ for BMI, which was higher than that used in previous studies [15, 16, 20]. This was probably linked to the increasing average BMI over the past 20 years [23, 32]. However, we observed a lower average BMI in the validation than in the derivation cohort, which implied that there was an improvement in health awareness in recent years.

However, this study had some limitations. First, it was a single-center design that enrolled a relatively homogenous population in both the derivation and validation cohorts. This might have limited the adaptability of the results to real-world situations. Second, the retrospective survey of the derivation cohort led to the loss of data to some extent, which resulted in potential selection bias. However, because the demographic features of the enrolled patients are similar to those of previous studies, such data limitations are likely minimal. Moreover, we performed a prospective validation cohort study to minimize this bias. Finally, the c-statistics for risk predictors of A-APCS in the validation cohort were not statistically significant compared to the APCS score; however, the P value (0.0593) was slightly higher than 0.05. Furthermore, the comparison of the c-statistic of the A-APCS score with that of the APCS in the derivation cohort showed that it was higher for A-APCS (P = 0.0056), implying that the A-APCS score presumably has a higher discriminatory ability for ACN risk-stratification. Additionally, we assessed the agreement of two scoring systems using Cohen’s Kappa Statistic, the risk stratification was better than the score of each patient, implying that the two systems had a “fair-moderate” level of agreement.

## Conclusion

This study updated the APCS score for prediction of ACN risk by applying data from two independent asymptomatic populations in China. Further research should evaluate the scoring model in clinical practice and community settings in other parts of the country.

## Data Availability

The datasets used and/or analysed during the current study available from the corresponding author on reasonable request.

## Abbreviations

A-APCS: adjusted Asia-Pacific colorectal screening
ACN: advanced colorectal neoplasia
APCS: Asia-Pacific Colorectal Screening
AR: average risk
BMI: body mass index
CI: confidence interval
CRC: colorectal cancer
FDR: first degree relatives
HGD: high-grade dysplasia
HR: high risk
ME-NBI: magnifying endoscopy with narrow band imaging
MR: moderate risk
OR: odds ratio
ROC: receiver operating characteristic
SSLs: sessile serrated lesions
